# Vitamin D Can Ameliorate Chlorhexidine Gluconate-Induced Peritoneal Fibrosis and Functional Deterioration through the Inhibition of Epithelial-to-Mesenchymal Transition of Mesothelial Cells

**DOI:** 10.1155/2015/595030

**Published:** 2015-10-01

**Authors:** Yi-Che Lee, Shih-Yuan Hung, Hung-Hsiang Liou, Tsun-Mei Lin, Chu-Hung Tsai, Sheng-Hsiang Lin, Yau-Sheng Tsai, Min-Yu Chang, Hsi-Hao Wang, Li-Chun Ho, Yi-Ting Chen, Ching-Fang Wu, Ho-Ching Chen, Hsin-Pao Chen, Kuang-Wen Liu, Chih-I. Chen, Kuan Min She, Hao-Kuang Wang, Chi-Wei Lin, Yuan-Yow Chiou

**Affiliations:** ^1^Division of Nephrology, Department of Internal Medicine, E-DA Hospital, I-Shou University, Kaohsiung, Taiwan; ^2^Institute of Clinical Medicine, National Cheng Kung University, Tainan, Taiwan; ^3^School of Medicine for International Students, I-Shou University, Kaohsiung, Taiwan; ^4^Division of Nephrology, Department of Medicine, Hsin-Jen Hospital, New Taipei City, Taiwan; ^5^Department of Laboratory Medicine, E-DA Hospital, I-Shou University, Kaohsiung, Taiwan; ^6^Division of Colorectal Surgery, Department of Surgery, E-DA Hospital, I-Shou University, Kaohsiung, Taiwan; ^7^Department of Neurosurgery, E-DA Hospital, I-Shou University, Kaohsiung, Taiwan; ^8^Department of Medical Education, I-Shou University, Kaohsiung, Taiwan; ^9^Department of Pediatrics, National Cheng Kung University Hospital, Tainan, Taiwan

## Abstract

*Background*. Peritoneal dialysis (PD) can induce fibrosis and functional alterations in PD patients' peritoneal membranes, due to long-term unphysiological dialysate exposure, partially occurring via triggering of epithelial-to-mesenchymal transition (EMT) in peritoneal mesothelial cells (MCs). Vitamin D can ameliorate these negative effects; however, the mechanism remains unexplored. Therefore, we investigated its possible links to MCs EMT inhibition. *Methods*. Peritoneal fibrosis was established in Sprague-Dawley rats by chlorhexidine gluconate (CG) intraperitoneal injection for 21 days, with and without 1*α*,25(OH)_2_D_3_ treatment. Morphological and functional evaluation and western blot analysis of EMT marker were performed upon peritoneum tissue. *In vitro* study was also performed in a primary human peritoneal MC culture system; MCs were incubated with transforming growth factor-*β*1 (TGF-*β*1) in the absence or presence of 1*α*,25(OH)_2_D_3_. EMT marker expression, migration activities, and cytoskeleton redistribution of MCs were determined. *Results*. 1*α*,25(OH)_2_D_3_ ameliorated CG-induced morphological and functional deterioration in animal model, along with CG-induced upregulation of *α*-SMA and downregulation of E-cadherin expression. Meanwhile, 1*α*,25(OH)_2_D_3_ also ameliorated TGF-*β*1-induced decrease in E-cadherin expression, increase in Snai1 and *α*-SMA expression, intracellular F-actin redistribution, and migration activity *in vitro*. *Conclusion*. 1*α*,25(OH)_2_D_3_ can ameliorate CG-induced peritoneal fibrosis and attenuate functional deterioration through inhibiting MC EMT.

## 1. Introduction

Peritoneal dialysis (PD) has become an important renal replacement therapy in recent decades in end-stage renal disease (ESRD) patients as hemodialysis (HD) [1–4]. However, the biggest limitation of PD therapy is that many patients have to shift to HD after several years due to treatment failure [5–9]. This failure is mainly attributed to inadequate dialysis, recurrent peritonitis, or peritoneal fibrosis [5, 6, 10, 11]. Patients' peritonitis rate and solute clearance have been greatly improved in recent years; however, PD-related peritoneal damage and fibrosis have become a major cause of treatment failure [11–13].

Under PD therapy, conventional bioincompatible dialysate, characterized by possessing acidic PH, hypertonicity, high glucose, and containing lactate, glucose degradation products (GDPs) induce mesothelial cell (MC) injury and promote pathological changes including induction of the epithelial-to-mesenchymal transition (EMT) process [14–16]. Subsequently, the peritoneal membrane undergoes several structural and functional changes, including fibrosis and neoangiogenesis, resulting in the promotion of peritoneal membrane failure [14, 15, 17, 18].

EMT plays a role in PD-related peritoneal membrane morphological and functional alterations [19, 20]. The EMT begins with the downregulation of selected adhesion molecules, such as E-cadherin, and dissociation of the intercellular junctions of MCs. Then, the cell cytoskeleton undergoes reorganization, and MCs acquire mesenchymal markers such as Snail and *α*-smooth muscle actin (*α*-SMA) [19]. Through this process, MCs gain higher invasive and migration capacities and promote peritoneal membrane fibrosis and functional changes [21, 22].

Recently, several studies have proven a protective effect of vitamin D against peritoneal fibrosis [23–27]. Furthermore, studies in the field of cancer research also found that vitamin D can ameliorate cancer cell EMT through the promotion of E-cadherin expression [28]. Therefore, in our study we aimed to investigate whether inhibition of the EMT process in MCs plays a role in the protective effects of vitamin D, both* in vitro* and* in vivo*.

## 2. Materials and Methods

### 2.1. Human Primary Mesothelial Cells

Omentum-derived MCs were obtained from nonuremic patients undergoing abdominal surgery; the omentum samples were digested in 0.05% trypsin and 0.02% EDTA to isolate MCs [14, 29]. All MCs isolated from the omentum were then incubated in culture medium consisting of Earle M199 medium (Gibco, NY, USA) supplemented with 10% fetal calf serum (Biological Industries, Kibbutz Beit-Haemek, Israel), insulin-transferrin-selenium-sodium pyruvate (ITS-A) (Gibco), 100 *μ*g/mL of streptomycin (MDBio, Inc., Taipei, Taiwan), 63.6 *μ*g/mL of penicillin G (Sigma-Aldrich, MO, USA), and 250 ng/mL Fungizone (MDBio, Inc.).

### 2.2. *In Vitro* Study Design

To investigate the effects of vitamin D on MC EMT process, omentum-derived MCs were treated with human recombinant transforming growth factor-*β*1 1 ng/mL (TGF-*β*1) (PeproTech, Rehovot, Israel) in culture medium with or without 1*α*,25(OH)_2_D_3_ (10^-6 ^mol/L, Sigma-Aldrich), the active form of vitamin D_3_.

### 2.3. Flow Cytometry and Immunofluorescence

To validate the purity of isolated MCs, omentum-derived cells were verified by ICAM-1 expression (anti-ICAM-1; eBioscience, CA, USA), determined according to a previously published protocol [14]. To validate changes in the EMT process, phalloidin-labeled staining (Life Technologies, NY, USA) and *α*-SMA staining (Sigma-Aldrich) were carried out [30]. DAPI staining was conducted for visualization of cell nuclei (Sigma-Aldrich).

### 2.4. Reverse Transcription-Polymerase Chain Reaction (RT-PCR)

Total RNA was extracted by using TRIzol method (Invitrogen, NY, USA) and complementary DNA was subsequently acquired from 1 *μ*g of total RNA by reverse transcription as per the manufacturer's instructions (Bio-Rad, CA, USA). E-cadherin mRNA was amplified by a Light Cycler using an SYBR Green Kit (Bio-Rad) and the specific primer set: 5′-GCATTGCCACATACACTCTCTTCT-3′ and 5′-CATTCTGATCGGTTACCGTGATC-3′. Snail mRNA was amplified using the primers 5′-GCAAATACTGCAACAAGG-3′ and 5′-GCACTGGTACTTCTTGACA-3′ under similar conditions. GAPDH was amplified using commercially produced primers: 5′-TGAACGGGAAGCTCACTGG-3′ and 5′-TCCACCACCCTGTTGCTGTA-3′. The annealing temperature used for all targets was 60°C. All samples were normalized to GAPDH.

### 2.5. Western Blotting for E-Cadherin and Snail

To investigate changes in MC EMT, primary antibodies to the epithelial marker, E-cadherin (mouse anti-E-cadherin, diluted 1 : 500; BD Bioscience, MA, USA), and the mesenchymal marker, Snail (rabbit anti-Snail; diluted 1 : 2000; Cell Signaling Technology Inc., MA, USA), were used [31, 32]. Each of the primary antibody blots was incubated with goat anti-mouse IgG (H+L) secondary antibody-HRP conjugate, diluted 1 : 5000 (Pierce, IL, USA), or goat anti-rabbit IgG (H+L) secondary antibody-HRP conjugate, diluted 1 : 5000 (KPL # 4741516), and visualized using enhanced chemiluminescence (Merck Millipore, Darmstadt, Germany).

### 2.6. Cell Migration Assay

MCs undergoing EMT show increased migration capacity; therefore, a wound-healing assay was performed to validate the response and change in EMT to 1*α*,25(OH)_2_D_3_. MCs were seeded at a concentration of 5 × 10^5^ in 6 cm culture dish. A scratch was made in the MC cell monolayer using a 200 *μ*L plastic pipette tip. MCs were then washed with PBS to remove the dead cells. The width of cell-free space was measured after the initial scratch at 0, 3, 6, 9, and 12 hours using a microscope (ZEISS; Primo Vert).

### 2.7. Quantification of *α*-SMA Positive Cells by Immunofluorescence

The *α*-SMA positive MCs were measured and expressed as mean ± standard deviation (S.D.). Each treatment group was measured at 10 random sites (magnification ×200) by blinded researchers using microscope analysis with a metric ocular.

### 2.8. Peritoneal Fibrosis-Model Experimental Protocol

To investigate the protective effects of 1*α*,25(OH)_2_D_3_ on peritoneal fibrosis, a total of 30 male Sprague-Dawley (SD) rats weighing 200 to 250 g were used. In the control group, rats received intraperitoneal (IP) injection of 1 mL/kg PBS in the first 7 days and then 1 mL/kg PBS followed by an additional 2 mL saline IP injection daily for a further 21 days (*N* = 6). Peritoneal fibrosis was induced by chlorhexidine gluconate (CG), as described previously by Coles and Topley [33]. Briefly, CG group rats received an IP injection of PBS (1 mL/kg) in the first 7 days and then PBS (1 mL/kg) followed by IP injection of 0.1% CG in ethanol (15%) dissolved in 2 mL saline daily for another 21 days (*N* = 6). In the group receiving CG with low dose vitamin D, rats received daily IP injections of 500 ng/kg vitamin D (Nang Kuang; Taiwan) in the first 7 days and then 500 ng/kg vitamin D followed by CG IP injection daily for another 21 days (*N* = 6). In the CG-receiving group with middle or high dose vitamin D, rats received IP injection of 750 ng/kg or 1 *μ*g/kg vitamin D, respectively, daily in the first 7 days and then the same dosage of vitamin D followed by CG IP injection daily for another 21 days (each groups *N* = 6). Finally, a modified peritoneal equilibration test was performed 29 days after the first IP injection and a blood sample was obtained by cardiac puncture [34]. The peritoneum and aorta were then removed by dissection.

### 2.9. Modified 4-Hour Peritoneal Equilibration Test

For evaluation of the peritoneal ultrafiltration rate, rats were anesthetized (Zoletil 50: Rompun 2% injection = 1 : 2; 100 *μ*L/100 g; intramuscular injection) and instilled with 90 mL/kg commercial dialysis solution containing 4.25% glucose (Dianeal; Baxter International, Inc., IL, USA), and 4 hr later, the rats were sacrificed by cervical dislocation to record the residual intraperitoneal volumes. Net ultrafiltration was calculated using the following formula: (final dialysate volume − initial dialysate volume)/initial dialysate volume. Glucose transport was obtained using the following formula: (initial dialysate glucose × initial volume) − (final dialysate glucose × final volume) [34].

### 2.10. Histopathological Examination

For histological analysis, 3 *μ*m thick paraffin sections of the rats' abdominal wall were stained by Masson's trichrome. The thickness of submesothelial tissue of the peritoneum was then measured and expressed as the mean ± S.D. For each rat, corresponding samples were measured at 6 random sites by blinded researchers performing microscope analysis with a metric ocular.

### 2.11. Quantification of Aortic Calcium and Phosphate

Aortic segments were lyophilized and decalcified with 0.6 N HCl and incubated at 37°C for 1 day. The o-cresolphthalein complexone kit (Teco Diagnostics, CA, USA) was used for determining the calcium content of the supernatant. The inorganic phosphorus reacted with ammonium molybdate in the sulfuric acid to form an unreduced phosphomolybdate complex. Then the complex absorbs light and was quantified photometrically in ultraviolet light. Light absorbance of the sample was directly proportional to the phosphorus concentration. Aortic calcium and phosphate content were normalized to the tissue dry weight (mg/g dry weight) [35].

### 2.12. Statistical Analysis

All data were expressed as mean ± S.D. and statistical significance was analyzed with a one-way analysis of variance. A significant result was defined as *P* < 0.05.

### 2.13. Ethics Statement

This study was approved by the ethics committee/institutional review board of E-Da Hospital, and written informed consent was obtained from all patients (IRB number: EMRP18100N).

## 3. Results

### 3.1. The Effect of Different Dosages of Vitamin D on Serum and Aortic Calcium and Phosphate Content

**Figure 1:**
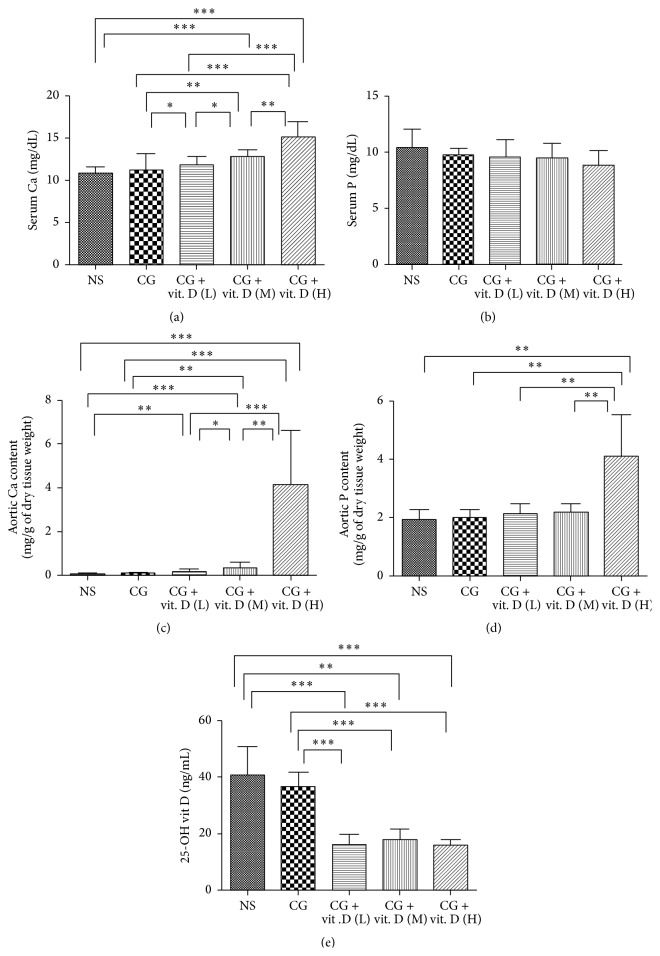
Effect of vitamin D on serum and aortic calcium and phosphate content. Sprague-Dawley rats received intraperitoneal (IP) injection of chlorhexidine gluconate (CG) daily with or without administration of low (L, 500 ng/kg), middle (M, 750 ng/kg), or high (H, 1 *μ*g/kg) dose 1*α*,25(OH)_2_D_3_ (vit. D). Rats also received daily intraperitoneal instillation of normal saline (NS) as a control. Blood samples and aorta tissue samples were taken 29 days after the first IP injection. (a) 1*α*,25(OH)_2_D_3_ induced mild hypercalcemia in the low and middle dose groups but induced severe hypercalcemia (>15 mg/dL) in the high dose group. (b) However, no significant difference was found in the serum phosphate content of the different groups. (c and d) High dose vitamin D also induced severe aortic calcium and phosphate deposition. (e) Further, we analyzed serum 25(OH)D levels, and the results indicated that endogenous 25(OH)D was significantly suppressed after 1*α*,25(OH)_2_D_3_ treatment. Data are represented as mean ± S.D. (^*∗*^ *P* < 0.05; ^*∗∗*^ *P* < 0.01; ^*∗∗∗*^ *P* < 0.005).

Vitamin D is well known to regulate serum calcium and phosphate and also vascular calcification. We therefore investigated the serum and aortic calcium and phosphate content in the rats under varying vitamin D dosage. 1*α*,25(OH)_2_D_3_ induced mild hypercalcemia in the low and middle dose groups but induced severe hypercalcemia (>15 mg/dL) in the high dose group, as shown in Figure 1(a). However, no significant difference was found in the serum phosphate content of the different groups (Figure 1(b)). High dose vitamin D also induced severe aortic calcium and phosphate deposition, as illustrated in Figures 1(c) and 1(d). Further, we analyzed serum 25(OH)D levels, and the results indicated that endogenous 25(OH)D was significantly suppressed after 1*α*,25(OH)_2_D_3_ treatment (Figure 1(e)).

### 3.2. Vitamin D Ameliorates CG-Induced Peritoneal Fibrosis in a Rat Model

**Figure 2:**
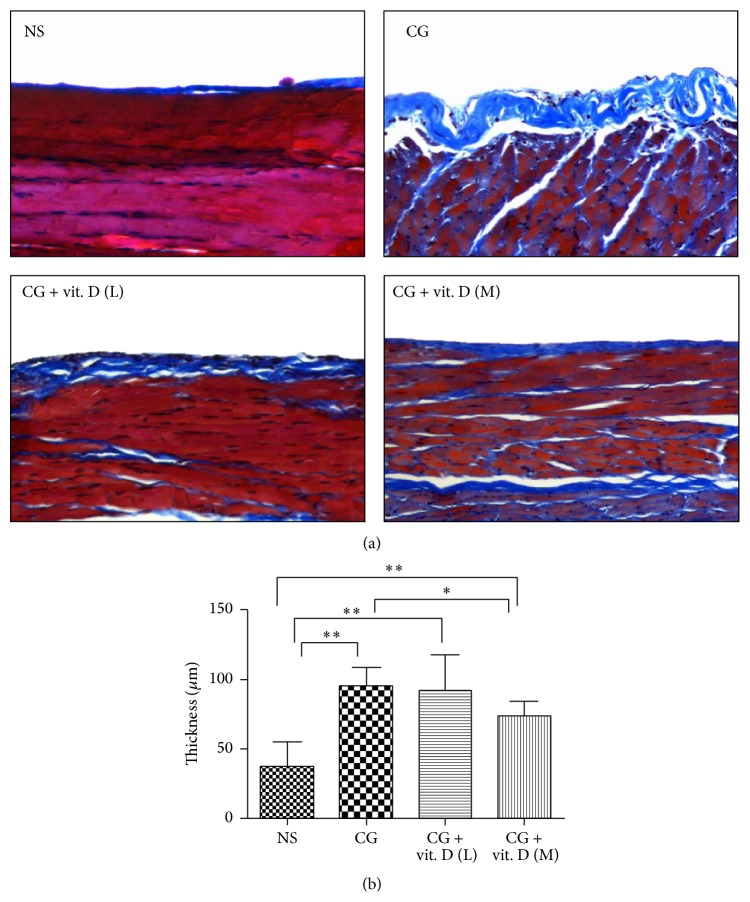
Vitamin D treatment decreased chlorhexidine gluconate- (CG-) induced peritoneal membrane fibrosis in a rat model. Sprague-Dawley rats received a daily intraperitoneal (IP) injection of chlorhexidine gluconate (CG) with or without administration of low (L, 500 ng/kg) or middle (M, 750 ng/kg) dose 1*α*,25(OH)_2_D_3_ (vit. D). Control rats received a daily IP instillation of normal saline (NS). (a) Representative images of peritoneum samples extracted 29 days after initial IP injection, stained with Masson's trichrome. CG instillation induced matrix deposition and thickening of the peritoneal membrane, while vitamin D treatment ameliorated these effects. Magnification ×200. (b) Quantification of the peritoneal membrane thickness. The antifibrotic effects of vitamin D were dose-dependent. Data represent mean ± S.D. (*n* = 6) (^*∗*^ *P* < 0.05; ^*∗∗*^ *P* < 0.01).

We then investigated whether vitamin D could ameliorate the structural deterioration of the peritoneal membrane in a peritoneal fibrosis animal model. As high dose vitamin D was demonstrated to induce severe hypercalcemia and aortic calcium and phosphate deposition, we postulated that 500 ng/kg (L) or 750 ng/kg (M) would be an ideal therapeutic dosage without apparent significant side effects. IP administration of 1*α*,25(OH)_2_D_3_ significantly ameliorated the peritoneal thickening in a dose-dependent manner as visualized by Masson's trichrome stain, shown in Figure 2.

### 3.3. Vitamin D Ameliorates CG-Induced Functional Deterioration of the Peritoneum in a Rat Model

**Figure 3:**
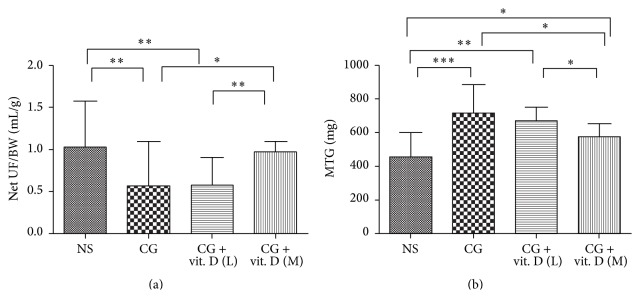
Vitamin D treatment prevented loss of peritoneal function caused by chlorhexidine gluconate (CG) exposure in a rat model. Sprague-Dawley rats received a daily intraperitoneal injection of chlorhexidine gluconate (CG) with or without administration of low (L, 500 ng/kg) or middle (M, 750 ng/kg) dose 1*α*,25(OH)_2_D_3_ (vit. D). Rats received a daily intraperitoneal instillation of normal saline (NS) as a control. Peritoneal function was assessed by (a) net ultrafiltration divided from body weight (UF/BW) and (b) mass transfer of glucose (MTG). CG instillation induced peritoneal function impairment, while vitamin D treatment significantly ameliorated this phenomenon. Data represent mean ± S.D. (*n* = 6) (^*∗*^ *P* < 0.05; ^*∗∗*^ *P* < 0.01; ^*∗∗∗*^ *P* < 0.005).

To further investigate the functional relevance of the peritoneum, a modified peritoneal equilibration test was performed on the final treatment day. The ultrafiltration volumes from the CG-exposed group were significantly lower than those from the saline group; and vitamin D was able to ameliorate CG-induced decrease in ultrafiltration, illustrated in Figure 3(a). In addition, the mass transport of glucose also indicated that vitamin D could ameliorate CG-induced increase in peritoneal permeability in a dose-dependent manner (Figure 3(b)).

### 3.4. Vitamin D Treatment Decreases CG-Induced EMT in a Rat Model

**Figure 4:**
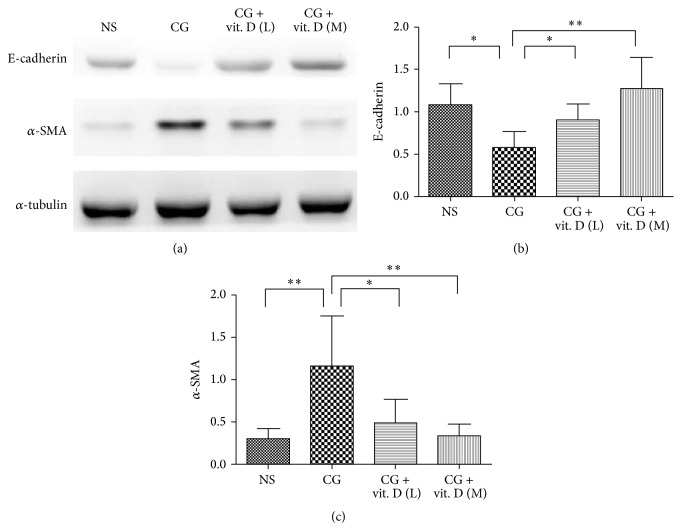
Vitamin D treatment decreased chlorhexidine gluconate- (CG-) induced peritoneal epithelial-to-mesenchymal transition in a rat model. Sprague-Dawley rats received a daily intraperitoneal injection of chlorhexidine gluconate (CG) with or without administration of low (L, 500 ng/kg) or middle (M, 750 ng/kg) dose 1*α*,25(OH)_2_D_3_ (vit. D). As a control, rats received daily intraperitoneal instillation of normal saline (NS). (a) Western blot analysis showed that vitamin D inhibited chlorhexidine gluconate- (CG-) induced downregulation of (b) E-cadherin and upregulation of (c) *α*-smooth muscle actin (*α*-SMA) in rat visceral peritoneum. Protein levels are represented semiquantitatively by the corresponding graphs and were measured on an arbitrary scale in both graphs. Data represent mean ± S.D. (*n* = 6) (^*∗*^ *P* < 0.05; ^*∗∗*^ *P* < 0.01).

We investigated the inhibitory effect of vitamin D on the EMT process* in vivo.* Western blot analysis showed that vitamin D inhibited CG-induced upregulation of *α*-SMA and downregulation of E-cadherin in rat visceral peritoneum (Figure 4).

### 3.5. Vitamin D Attenuates TGF-*β*-Induced EMT and Migration Activity* In Vitro*

**Figure 5:**
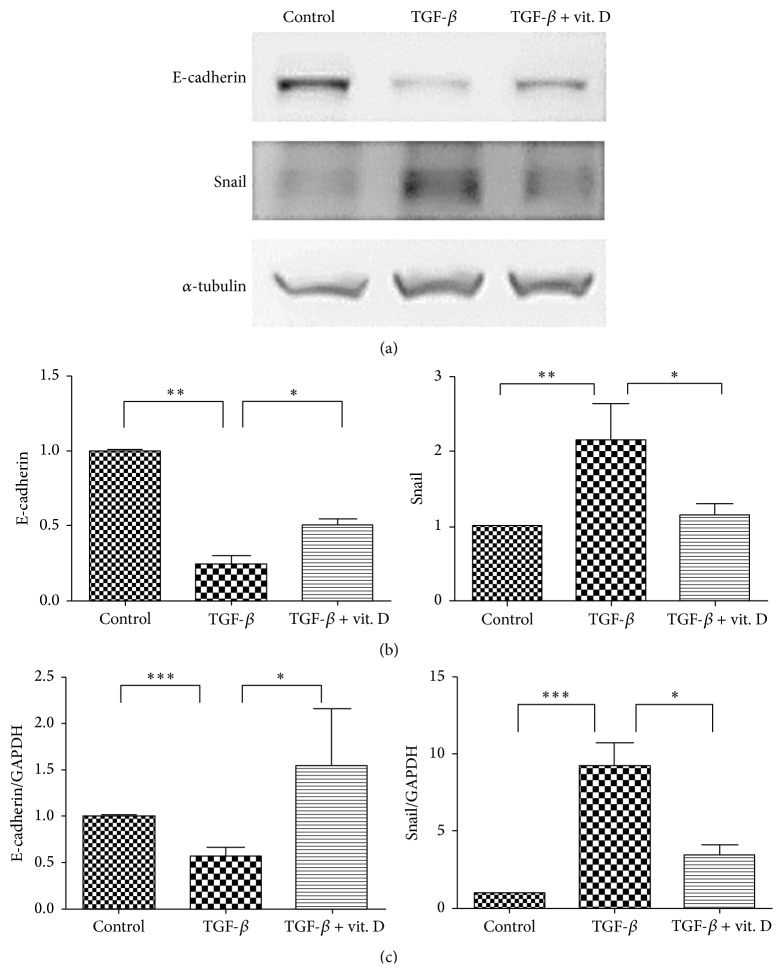
Vitamin D3 inhibited transforming growth factor-*β*1- (TGF-*β*1-) induced epithelial-to-mesenchymal transition (EMT) of mesothelial cells (MCs)* in vitro*. (a) Primary human peritoneal MCs were incubated with TGF-*β*1* in vitro*. Western blot analysis showed that addition of 1*α*,25(OH)_2_D_3_ (vit. D, 10^−6^ mol/L) inhibited TGF-*β*1-induced EMT (epithelial marker: E-cadherin; mesenchymal marker: Snail). (b) Semiquantitative data of protein levels of E-cadherin and Snail. (*N* = 6) (c) Vitamin D inhibited TGF-*β*1-induced downregulation of E-cadherin and upregulation of Snail mRNA (normalized with GAPDH). Protein levels were measured on an arbitrary scale in all graphs (protein level from Western blots was performed using AlphaImager 2200). Data are represented as mean ± S.D. (*N* = 3) (^*∗*^ *P* < 0.05; ^*∗∗*^ *P* < 0.01; ^*∗∗∗*^ *P* < 0.005).

**Figure 6:**
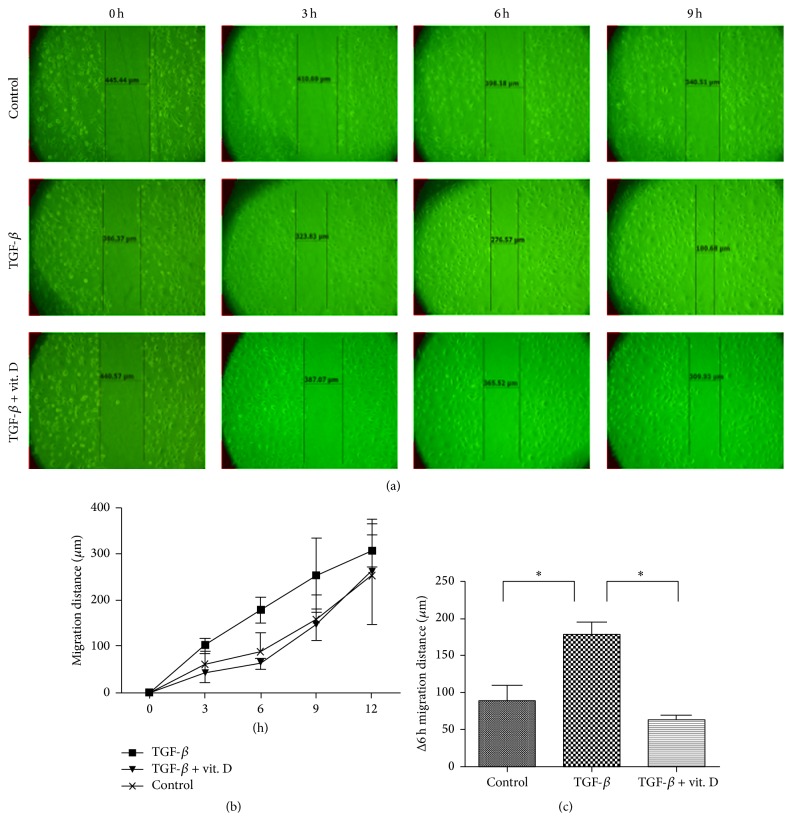
Vitamin D3 inhibited transforming growth factor-*β*1- (TGF-*β*1-) induced migration activity of mesothelial cells (MCs)* in vitro*. (a) Representative images of MCs in the wound-healing assay. MCs were treated with TGF-*β*1 (1 ng/mL) with or without 1*α*,25(OH)_2_D_3_ (vit. D, 10^−6^ mol/L). (b) Quantitative analysis of the width of the postscratch cell-free space at each follow-up time point. (c) The difference in the migration distance at 6 hr (Δ6 hr) is represented in (c). TGF-*β*1 promoted MCs migration activity was inhibited by vitamin D3. (*N* = 4) Graphical data represent mean ± S.D. (^*∗*^ *P* < 0.05).

**Figure 7:**
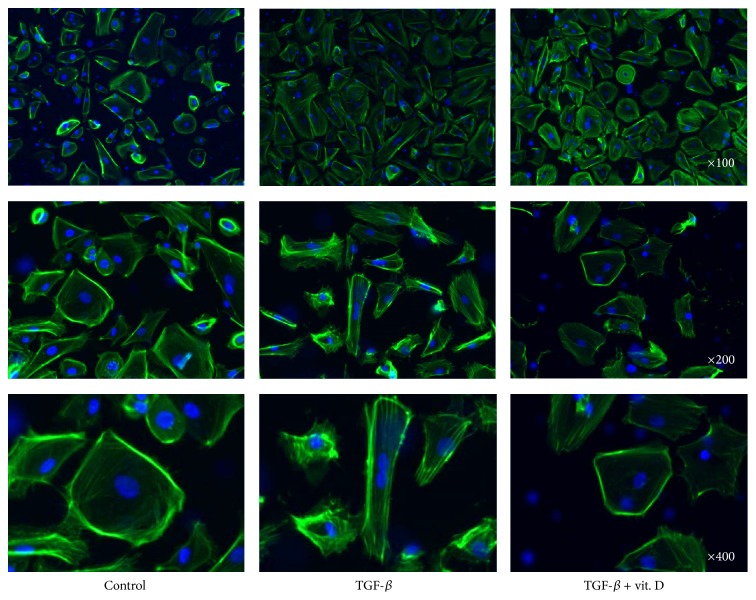
Vitamin D3 inhibited transforming growth factor-*β*1- (TGF-*β*1-) induced mesothelial cell (MC) cytoskeleton redistribution* in vitro*. MCs were cultured with TGF-*β*1 with or without addition of 1*α*,25(OH)_2_D_3_ (vit. D). MC cytoskeletons were stained with anti-F-actin antibodies. In normal culture medium, the actin cytoskeleton (green) was in the cortical band and TGF-*β*1 promoted cytoskeleton redistribution (fibroblast-like cytoskeleton). Vitamin D inhibited TGF-*β*1-induced cytoskeleton redistribution. Nuclei were stained with DAPI (blue). Magnification ×400.

**Figure 8:**
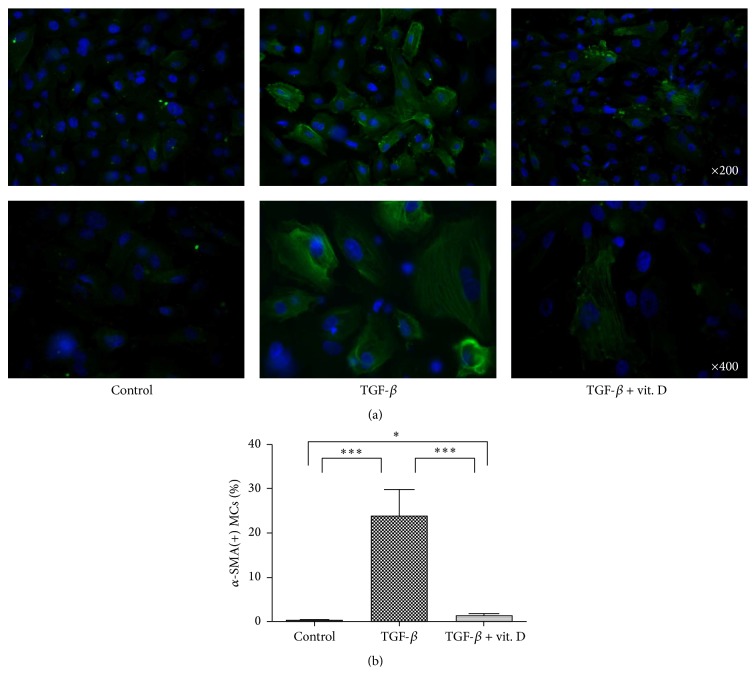
Vitamin D3 inhibits transforming growth factor-*β*1- (TGF-*β*1-) induced *α*-smooth muscle actin (*α*-SMA) upregulation of mesothelial cells (MCs)* in vitro*. MCs were treated with TGF-*β*1 with or without addition of 1*α*,25(OH)_2_D_3_ (vit. D). Immunofluorescence analysis showed that TGF-*β*1 promoted upregulation of *α*-SMA (green) in MCs and vitamin D inhibited this phenomenon. Nuclei are stained with DAPI (blue). This inhibition effect is quantified in (b) where the positive staining for *α*-SMA was calculated as a percentage of MCs present. Data represent mean ± S.D. (^*∗*^ *P* < 0.05; ^*∗∗∗*^ *P* < 0.005).

To further confirm that vitamin D exerts its antifibrotic effect through inhibition of the EMT process of MCs, primary human MCs were incubated with TGF-*β*1 in the absence or the presence of vitamin D. RT-PCR and western blot analysis both verified that 1*α*,25(OH)_2_D_3_ significantly ameliorated TGF-*β*1-induced EMT (Figure 5). We subsequently investigated the effect of vitamin D on cell migration activity as cells gain greater migration ability after undergoing EMT. The results showed that 1*α*,25(OH)_2_D_3_ significantly inhibited TGF-*β*1 promoted cell migrating activity (Figure 6). Furthermore, we also verified the change in the EMT process by phalloidin-labeled staining to visualize cytoskeletal actin expression changes in MCs. As evidenced in Figure 7, 1*α*,25(OH)_2_D_3_ inhibited TGF-*β*1-induced intracellular F-actin redistribution. TGF-*β*1 promoted upregulation of *α*-SMA in MCs was also demonstrated (Figure 8) and it was found that vitamin D could inhibit this effect.

## 4. Discussion

Our data showed that 1*α*,25(OH)_2_D_3_ ameliorates CG-induced morphological and functional deterioration of the peritoneal membrane in a dose-dependent manner and that vitamin D could inhibit the CG-induced EMT process* in vivo*. In addition, our* in vitro* study also proved that vitamin D inhibited TGF-*β*1-induced EMT marker change, migrating activities, and cytoskeleton redistribution in MCs. Taken together, our study demonstrated that inhibition of the EMT process of MCs plays a role in the peritoneum-protective effect of vitamin D.

Several studies have previously investigated the protective effect of vitamin D on peritoneal membranes. Coronel et al. reported a preliminary study showing that paricalcitol decreased peritoneal membrane permeability, with diminished peritoneal protein loss and increased ultrafiltration in PD patients [23]. Lee et al. proved that calcitriol could decrease peritoneal fibrosis in a rat model of acute CG exposure. Further, expression of TGF-*β*1 and angiotensin II induced by CG were also reduced [27]. González-Mateo et al. reported that paricalcitol could ameliorate peritoneal fibrosis in a murine PD model through the activation of regulatory T cells and reduction in IL-17 production [25]. Recently, Kang et al. showed that paricalcitol affected EMT [26]. However, there were some limitations to their study. First, the* in vitro* study did not completely validate the EMT process. They only investigated the expression of EMT markers but not the migration capacity and cytoskeletal redistribution of MCs. Second, in their* in vivo* study, they only investigated the kinetics of glucose mass transfer but not of ultrafiltration. In addition, they only examined the expression of the mesenchymal marker *α*-SMA in the peritoneum; no epithelial marker was analyzed. Finally, the study lacked information about the influence of their therapeutic dose of vitamin D on serum calcium and phosphate levels and vascular calcification and the dose-dependent effects of therapeutic vitamin D were also unknown.

TGF-*β*1 is a key cytokine for the promotion of EMT in peritoneal MCs and of peritoneal fibrosis [36]. TGF-*β*1 binds to its receptor and exerts its biological and pathological activities via activation of Smad and non-Smad signaling pathways [37]. The Smad-dependent signaling pathway is the major mechanism of EMT and fibrosis through promotion of mesenchymal marker overexpression, such as *α*-SMA and Snail, and suppression of epithelial markers, such as E-cadherin and cytokeratin. There are several possible mechanisms that explain how vitamin D inhibits MC EMT. Firstly, downregulation of E-cadherin is an important step in EMT and vitamin D has been proven to promote E-cadherin expression in some cancer studies [28]. Secondly, under MC injury conditions, MCs will express and secrete TGF-*β*1; TGF-*β*1 then channels back to induce MC EMT. At least one study had proven that vitamin D can inhibit TGF-*β*1 expression and secretion during MC damage [27]. Besides, inhibition of MC EMT by vitamin D may be also attributed to the inhibitory effects on TGF-*β* dependent pathways. For example, Nolan et al. report that vitamin D significantly attenuated TGF-*β*1-induced renal epithelial injury* in vitro* through attenuating Smad2 phosphorylation [38]. Zerr et al. also proved that vitamin D receptor regulates Smad3-dependent transcription [39].

Our study had several limitations. The first was the use of CG as a chemical irritant and not dialysate to induce peritoneal fibrosis and functional deterioration in our animal model. Furthermore, our model did not contain uremic rats; uremic toxins effect on peritoneum could therefore not be measured here. The CG model of peritoneal fibrosis is a simple model and is easy to use but certainly may not be an ideal substitute for PD fluid installation [40]. It should be emphasized that results obtained in CG models may not translate to PD fluid models of fibrosis as in the latter the changes are more subtle and may follow different pathways of fibrosis. However, previous studies have proved that it is a feasible model for studying peritoneal fibrosis [41–43]. Second, although our data suggest that vitamin D can ameliorate peritoneal membrane morphological and functional deterioration, the results also showed that vitamin D may induce a mild degree of vascular calcification and hypercalcemia under our therapeutic dosage. Besides, in the human setting, 1-2 *μ*g of 1*α*,25(OH)_2_D_3_ in a daily dose is considered a high dose and may already induce hypercalcemia. But in our experimental model, the dosage was at least ten times more than human setting and really a supraphysiological dose. Further study is required to resolve this problem and we postulate that a tissue-specific drug delivery system may be an ideal solution [44]. Finally, many previous studies have shown MCs to be an important source of myofibroblasts via the EMT process [14, 19, 45]; however, one recent* in vivo* study did suggest that MCs may not be the main source of submesothelial fibroblasts [46]. Additional study is needed to confirm this result; but we hypothesize that even if EMT MCs are not the main source of submesothelial fibroblasts, they will still play a role in the process of fibrosis.

## 5. Conclusions

The present study demonstrates that vitamin D can ameliorate EMT process in MCs, improve peritoneal fibrosis, and attenuate functional deterioration. We propose that vitamin D may be an advantageous addition to PD therapy, preserving peritoneum function during long-term PD and preventing technique failure.

## Abbreviations

*α*-SMA: *α*-smooth muscle actin
CG: Chlorhexidine gluconate
EMT: Epithelial-to-mesenchymal transition
ESRD: End-stage renal disease
GDPs: Glucose degradation products
HD: Hemodialysis
ICAM-1: Intercellular adhesion molecule-1
IP: Intraperitoneal
MCs: Mesothelial cells
PBS: Phosphate buffered saline
PD: Peritoneal dialysis
SD: Sprague-Dawley
S.D.: Standard deviation
TGF-*β*1: Transforming growth factor-*β*1.
